# Hormonal and Cytokine Imbalances Promote a Proinflammatory Profile in Institutionalized Elderly

**DOI:** 10.3390/brainsci15121310

**Published:** 2025-12-05

**Authors:** Arce dos Santos Sfredo, Marcondes Alves Barbosa da Silva, Laura Valdiane Luz Melo, Danielle Cristina Honorio França, Gabriel Lopes Dantas, Wagner Batista dos Santos, Danny Laura Gomes Fagundes-Triches, Patrícia Gelli Feres de Marchi, Adenilda Cristina Honorio-França, Eduardo Luzía França

**Affiliations:** 1 Postgraduate Program in Basic and Applied Immunology and Parasitology, Federal University of Mato Grosso, Barra do Garças 78600-000, Brazilpgfmarchi.ufmt@gmail.com (P.G.F.d.M.); adenilda.franca@ufmt.br (A.C.H.-F.); 2 Institute of Health Science, Federal University of Rondonópolis, Rondonópolis 78736-900, Brazil; 3 Department of Gynecology and Obstetrics, Botucatu Medical School (FMB), São Paulo State University (UNESP), Botucatu 18618-687, Brazil; danielle.franca@unesp.br; 4 Postgraduate Program in Materials Science, Araguaia University Campus, Federal University of Mato Grosso, Barra do Garças 78605-091, Brazil; 5 Institute of Biological and Health Science, Federal University of Mato Grosso, Barra do Garças 78600-000, Brazil

**Keywords:** cortisol, melatonin, cytokines, immunosenescence, inflammaging, institutionalized elderly

## Abstract

Background: Aging is accompanied by chronic low-grade inflammation (inflammaging) and neuroendocrine–immune imbalance. This study evaluated blood concentrations of cortisol, melatonin, and Th1/Th2/Th17 cytokines in elderly individuals. Methods: A cross-sectional study was conducted, including institutionalized elderly individuals (*n* = 32) and elderly individuals living with family members (*n* = 29). Blood samples were collected between 8:00 and 10:00 a.m. for cytokine quantification (IL-2, IL-4, IL-6, IL-10, IL-17, TNF-α, IFN-γ) by flow cytometry and for cortisol and melatonin measurement by ELISA, ensuring accurate interpretation while considering their circadian variations. Results: Institutionalized individuals showed higher IL-6 (*p* = 0.0261) and IFN-γ (*p* = 0.0065), and lower IL-2 (*p* = 0.0006), IL-4 (*p* = 0.0043), IL-17 (*p* = 0.0025), and TNF-α (*p* = 0.0243). Blood cortisol (*p* = 0.0309) and melatonin (*p* = 0.0407) were also elevated. Cortisol correlated negatively with IL-2 (*r* = −0.5986, *p* = 0.0397) and IL-6 (*r* = −0.6135, *p* = 0.0338). Conclusions: Institutionalization is associated with elevated blood hormone levels and an imbalanced cytokine pattern, indicating disruption of the neuroendocrine–immune network. These alterations align with the concept of inflammaging and highlight the impact of the living environment on immune–endocrine regulation in older adults.

## 1. Introduction

Aging is accompanied by chronic low-grade inflammation, collectively known as inflammaging, characterized by immune dysregulation, oxidative stress, and impaired clearance of senescent cells, contributing to the development of age-related diseases [1,2,3,4]. These alterations are markedly intensified in institutionalized elderly individuals, who experience higher rates of frailty, psychosocial stress, physical inactivity, multimorbidity, and polypharmacy compared with their community-dwelling counterparts [5,6,7]. Environmental conditions such as limited sunlight exposure, fragmented sleep–wake cycles, and reduced social interaction further contribute to immune vulnerability in this population [8]. Moreover, psychosocial and environmental stressors typical of long-term care settings, including social isolation, reduced environmental stimulation, and circadian disruption, have been associated with dysregulation of inflammatory mediators such as IL-6 and TNF-α, reinforcing the link between stress-related pathways and impaired immune control in aging [9,10]. Several studies comparing institutionalized older adults with community-dwelling peers have identified distinct physiological, inflammatory, and frailty-related profiles [11,12,13]. However, despite these advances, investigations specifically addressing how neuroendocrine mediators, particularly cortisol and melatonin, interact with Th1, Th2, and Th17 cytokine pathways in institutionalized settings remain limited. As the global population ages, the number of institutionalized older adults continues to rise, underscoring the clinical relevance of better understanding the health-related determinants in this demographic [14].

The neuroendocrine–immune axis plays a central role in maintaining physiological stability during aging. Cortisol, a glucocorticoid regulated by the hypothalamic–pituitary–adrenal (HPA) axis, modulates metabolic and inflammatory pathways through inhibition of NF-κB signaling and regulation of cytokine production [15,16,17]. Aging is associated with disrupted cortisol rhythms, elevated basal levels, and weakened HPA feedback, which contribute to frailty, cognitive decline, and impaired immune competence [18]. Melatonin, produced by the pineal gland in response to darkness, exerts antioxidant and immunomodulatory actions while synchronizing circadian rhythms [19,20,21]. Age-related melatonin decline exacerbated in institutionalized settings with irregular light exposure has been associated with oxidative stress, sleep disturbances, and altered cytokine secretion [3,22,23]. Disruption of circadian organization, common in long-term care facilities, has been linked to dysregulation of IL-6, TNF-α, and other cytokines, indicating a bidirectional relationship between neuroendocrine signaling and immune activity [24,25].

Despite growing evidence that Th1, Th2, and Th17 cytokines are key components of immunological aging, and that cortisol and melatonin critically regulate neuroendocrine–immune interactions under chronic low-grade inflammation, few studies have examined these relationships in institutionalized elderly individuals. Understanding this interplay is essential to elucidate how neuroendocrine–immune imbalance contributes to immune vulnerability in aging.

Therefore, this study aimed to assess blood levels of cortisol, melatonin, and Th1/Th2/Th17 cytokines in institutionalized elderly individuals and to compare them with those of community-dwelling peers. We hypothesized that institutionalization is associated with a distinct neuroendocrine–immune signature characterized by hormonal imbalance and altered cytokine profiles, providing new insights into mechanisms underlying immune vulnerability in older age.

## 2. Materials and Methods

### 2.1. Study Design and Participants

This study employs an analytical, quantitative approach with a cross-sectional design. The sample size was calculated using a significance level (α) of 0.05 and a power (β) of 0.20, which corresponds to a statistical power of 80%. Based on these parameters, a minimum of 20 individuals per group was required to ensure adequate sensitivity for detecting differences among variables.

The study was conducted in the municipalities of Barra do Garças (Mato Grosso, Brazil) and Aragarças (Goiás, Brazil), two neighboring cities located on opposite banks of the Araguaia River. The patients were recruited in 2023 and 2024. The Institutional Research Ethics Committee approved this study, and all participants provided written informed consent.

Institutionalized older adults were recruited from the Associação Beneditina da Providência long-term care facility in Aragarças, Goiás, while non-institutionalized participants were community-dwelling older adults enrolled in primary healthcare programs in Aragarças, GO, and Barra do Garças, MT, ensuring comparable cultural and socioeconomic backgrounds. The institutionalized participants resided in a long-term care facility with standardized daily routines. Wake-up times (6:00 a.m. to 7:00 a.m.), meal times, and medication administration were fixed and uniformly applied. Residents spent most of the day indoors, with limited exposure to natural sunlight, and access to the outdoors only during brief scheduled periods. None of the institutionalized elderly engaged in regular physical activity, and although only 21% had formal mobility restrictions (bedridden or wheelchair-bound), overall activity levels were remarkably low. These environmental and behavioral characteristics, particularly reduced exposure to natural light and minimal physical activity, are relevant contextual factors given their known influence on circadian, neuroendocrine, and immunological regulation.

A total of 74 individuals were initially screened; however, those with autoimmune diseases, decompensated metabolic conditions (including diabetes mellitus or severe obesity), acute inflammatory diseases, or extreme age (≥100 years) were excluded from the analysis.

After applying the inclusion and exclusion criteria, the final sample consisted of 61 participants, including 27 women and 34 men, divided into institutionalized (*n* = 32) and non-institutionalized (*n* = 29) groups. All participants were over 60 years of age.

**Scheme 1:**
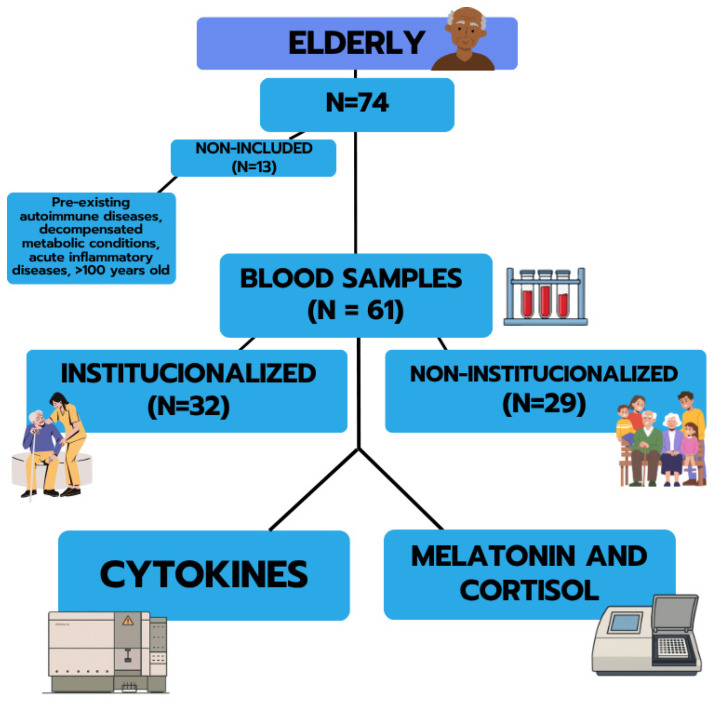
Flowchart representing the study design and sample distribution. Of the 74 elderly individuals initially, 13 were excluded due to pre-existing conditions or age over 100 years. Blood samples were obtained from 61 participants, divided into institutionalized (*n* = 32) and non-institutionalized (*n* = 29) groups. In elderly individuals, cytokine, melatonin, and cortisol levels were measured from both study groups.

The workflow for participant selection, sample collection, and experimental design is summarized in Scheme 1. All procedures were conducted in accordance with the Declaration of Helsinki, and written informed consent was obtained from all participants before enrollment.

### 2.2. Sample Collection

Blood samples were collected from institutionalized and non-institutionalized elderly individuals to evaluate cytokine profiles and hormone levels. All participants underwent overnight fasting (8–10 h), and collections were performed in the morning between 8:00 and 10:00 a.m., ensuring standardized conditions and allowing appropriate interpretation of cortisol and melatonin levels according to their circadian rhythms. Samples were obtained by venipuncture, with approximately 10 mL collected into Vacutainer tubes containing clot activator (Becton, Dickinson and Company, Franklin Lakes, NJ, USA). Serum was separated by centrifugation at 160× *g* for 10 min and immediately aliquoted to prevent degradation of thermosensitive analytes. Aliquots were stored at −80 °C for up to 6 months before cytokine and hormone quantification. To preserve analyte stability, all samples were thawed only once, and no aliquot underwent more than a single freeze–thaw cycle. All measurements were performed using first-thaw aliquots, avoiding the well-documented loss of cytokine integrity caused by repeated freeze–thaw events. All samples were coded, and laboratory personnel conducting the assays were blinded to participants’ group allocation to minimize analytical bias.

### 2.3. Cytokines Determination

Cytokine levels in serum from elderly individuals, institutionalized or not, were assessed using the Cytometric Bead Array (CBA) Human Th1/Th2/Th17 Cytokine Kit (BD Biosciences, San Jose, CA, USA). In this study, cytokines IFN-γ, TNF-α, IL-2, IL-4, IL-6, IL-10, and IL-17 were quantified in serum samples. Measurements were performed on a FACSCalibur flow cytometer (BD Bioscience, San Jose, CA, USA). Instrument settings were verified with BD Calibrite™ 3 tracking beads, and compensation was established using BD Calibrite™ 3 compensation beads according to the manufacturer’s instructions. The same compensation matrix was applied to all samples.

A standardized gating strategy appropriate for the Th1/Th2/Th17 CBA kit was applied. First, bead populations were isolated based on characteristic forward- and side-scatter (FSC × SSC) signals, separating bead events from debris. Next, individual cytokine-specific bead regions (A–G) were discriminated using their distinct APC fluorescence signatures detected in FL3, as defined by the kit’s capture bead profiles. For each bead region, PE fluorescence (FL2) corresponding to the bound detection reagent was quantified as a measure of cytokine concentration. Only singlet bead events were included in the analysis. A minimum of 3000 bead events per analyte was acquired to ensure analytical robustness.

The lower limits of detection (LOD) for each analyte, as reported by the manufacturer, were IL-2 = 2.6 pg/mL, IL-4 = 2.6 pg/mL, IL-6 = 3.0 pg/mL, IL-10 = 2.8 pg/mL, TNF-α = 3.7 pg/mL, IFN-γ = 3.8 pg/mL, and IL-17A = 18.9 pg/mL. Samples with concentrations below the detection threshold were considered below detection limit (BDL) and assigned the lowest detectable value. All samples were processed and analyzed under identical conditions to minimize technical variability. Data were analyzed using BD FCAP Array^TM^ software (version 3.0).

### 2.4. Melatonin and Cortisol Determination

Serum melatonin levels were determined using a commercial ELISA kit (Immuno-Biological Laboratories [IBL], Hamburg, Germany) according to the manufacturer’s protocol. Before analysis, melatonin was extracted from serum samples using standardized affinity chromatography columns to remove interfering components. The assay presented a lower detection limit of 1.6 pg/mL, with intra-assay and inter-assay coefficients of variation (CVs) ranging from 3.0% to 11.4% and 6.4% to 19.3%, respectively.

After extraction, each sample, standard, and control was pipetted onto a 96-well microplate and incubated according to the manufacturer’s recommendations. Absorbance was measured with a microplate spectrophotometer at 405 nm, and concentrations were calculated from a standard calibration curve. The results were expressed in pg/mL.

Serum cortisol concentrations were quantified using a microplate ELISA kit (DRG^®^ International, Inc., Springfield, NJ, USA) according to the manufacturer’s instructions. The assay exhibited a lower detection limit of 100 pg/mL, with intra-assay and inter-assay CVs of 8.1% and 8.8%, respectively. Standards, controls, and serum samples were processed under identical conditions, and absorbance was measured at 450 nm using a microplate spectrophotometer. Cortisol concentrations were calculated from a standard curve and expressed in ng/mL.

### 2.5. Statistical Analysis

Data are presented as mean ± standard deviation. The D’Agostino test was used to assess data normality. Comparisons between two independent groups were conducted using Student’s *t*-test. Differences in cytokine concentrations and hormones were analyzed. Correlations between hormones and cytokines were evaluated using Pearson’s linear correlation. Statistical significance was set at *p* < 0.05.

Effect sizes were calculated for all between-group comparisons using Cohen’s d, with values of 0.2, 0.5, and 0.8 interpreted as small, medium, and large effects, respectively. Post Hoc power analyses (1 − β) were conducted based on the observed effect sizes and final sample sizes after exclusions, using a two-tailed α = 0.05. Power was estimated with the noncentral t distribution for independent-samples *t*-tests.

## 3. Results

### 3.1. General Characteristics of Patients

Table 1 summarizes the anthropometric and clinical characteristics of the elderly participants included in the study, divided into non-institutionalized and institutionalized groups. Both groups were comparable with respect to sex distribution and metabolic parameters, with no significant differences in body weight, body mass index (BMI), waist circumference, or fasting glucose levels.

The mean age of institutionalized individuals (78.2 ± 8.1 years) was similar to that of the non-institutionalized group (74.4 ± 7.5 years). The proportion of female participants was similar in both groups (41.4% vs. 43.7%). The prevalence of hypertension (62.1% vs. 65.6%) and diabetes mellitus (20.7% vs. 21.8%) did not differ substantially between groups.

### 3.2. Cytokine Levels

**Figure 1:**
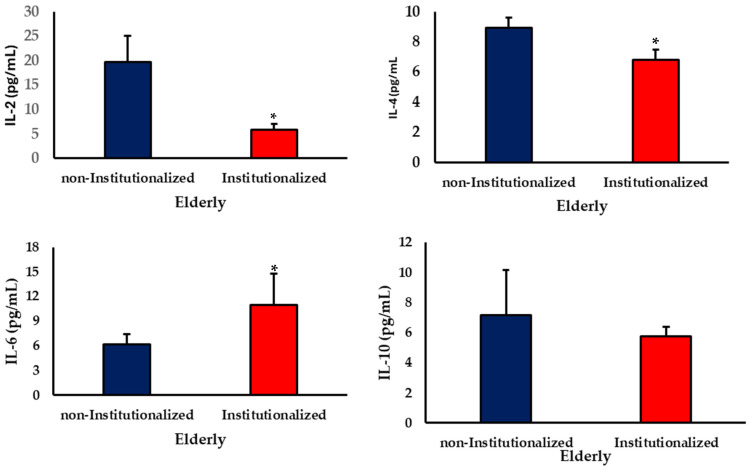

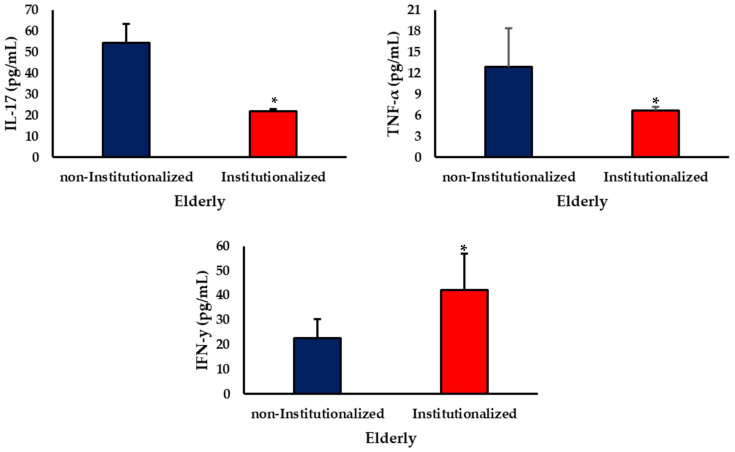
Cytokine profiles in institutionalized and non-institutionalized elderly individuals. Serum concentrations (pg/mL) of IL-2, IL-4, IL-6, IL-10, IL-17, TNF-α, and IFN-γ in institutionalized (*n* = 18) and non-institutionalized elderly individuals (*n* = 19). Data are presented as individual values with mean ± SE. Statistical analysis was performed using Student’s *t*-test. * indicates significant differences between groups: IL-2 (*p* = 0.0006), IL-4 (*p* = 0.0043), IL-6 (*p* = 0.0261), IL-10 (*p* = 0.1599), IL-17 (*p* = 0.0025), TNF-α (*p* = 0.0243), and IFN-γ (*p* = 0.0065).

Figure 1 shows the cytokine concentrations of the elderly participants. The analysis revealed marked differences between institutionalized and non-institutionalized individuals. IL-2 (5.9 ± 1.2), IL-4 (6.8 ± 0.7), IL-17 (21.9 ± 1.0), and TNF-α (6.7 ± 0.5) levels were significantly lower in institutionalized participants compared with the non-institutionalized group: IL-2 (19.5 ± 5.4), IL-4 (8.9 ± 0.6), IL-17 (54.3 ± 9.1), and TNF-α (12.9 ± 5.4). Conversely, IL-6 and IFN-**γ** concentrations were higher among institutionalized individuals (10.9 ± 3.8 and 42.0 ± 14.8, respectively) than among those living in the community (6.1 ± 1.3 and 27.7 ± 7.5, respectively). No significant difference was observed for IL-10, whose levels were similar between institutionalized (5.8 ± 0.6) and non-institutionalized participants (7.1 ± 3.0).

Effect size analysis revealed markedly large differences for most cytokines between institutionalized and non-institutionalized elderly individuals. IL-2 (|d| = 3.76), IL-4 (|d| = 3.18), and IL-17 (|d| = 5.44) showed extremely large effects, while IL-6 (|d| = 1.59), TNF-α (|d| = 1.76), and IFN-γ (|d| = 1.57) also demonstrated large effect sizes, all accompanied by high post hoc power (1 − β > 0.90). In contrast, IL-10 showed a moderate effect size (d = 0.65) and low statistical power (1 − β = 0.29), consistent with the absence of significant group differences.

**Figure 2:**
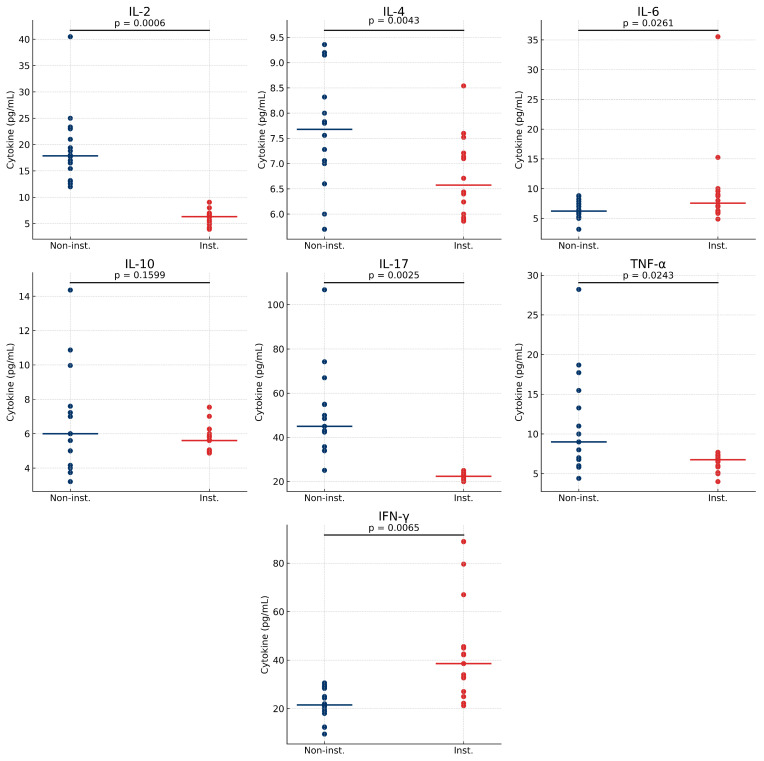
Individual cytokine distributions in institutionalized (Inst.) and non-institutionalized (Non-inst.) elderly individuals. Dot plots show serum concentrations (pg/mL) of IL-2, IL-4, IL-6, IL-10, IL-17, TNF-α, and IFN-γ. Each dot represents an individual participant; institutionalized elderly are shown in red, and non-institutionalized elderly in dark blue. Horizontal lines indicate group medians.

Figure 2 illustrates the comparative serum concentrations of cytokines IL-2, IL-4, IL-6, IL-10, IL-17, TNF-α, and IFN-γ between institutionalized and non-institutionalized elderly participants. The non-institutionalized group showed greater dispersion in most cytokines, particularly IL-2, IL-17, and TNF-α, indicating greater interindividual variability in immune responses. In contrast, the institutionalized group showed more compact distributions of these cytokines, except for IL-6 and IFN-γ, which showed greater dispersion and higher average values, consistent with a proinflammatory state. The median lines (dashed) clearly illustrate lower central tendencies for IL-2, IL-4, IL-17, and TNF-α in institutionalized individuals. In contrast, the medians for IL-6 and IFN-γ are shifted upward, reinforcing the pattern of immune imbalance characteristic of inflammaging.

**Figure 3:**
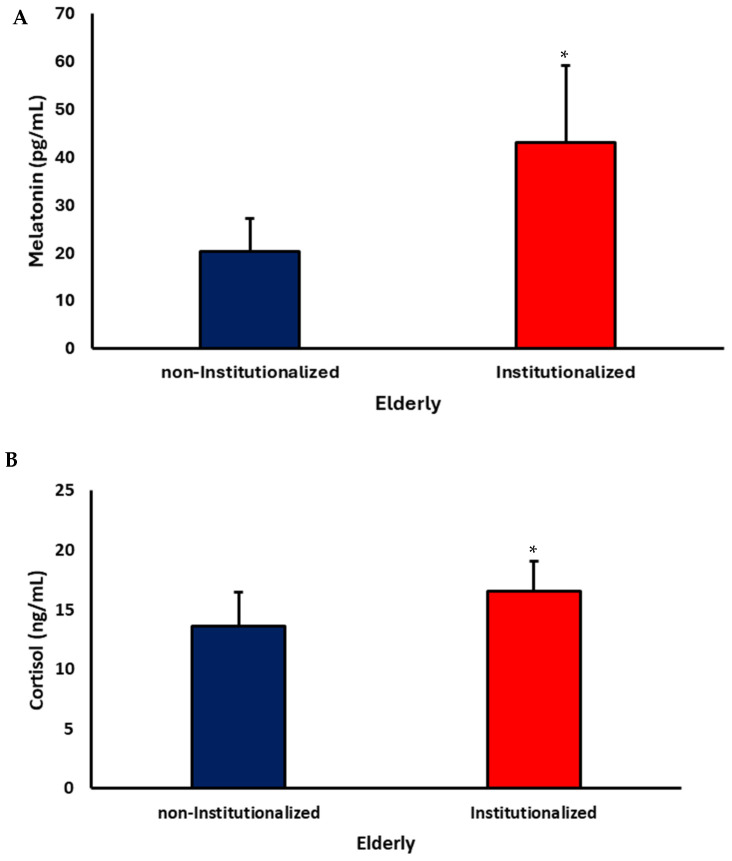
Serum hormone concentrations in institutionalized and non-institutionalized elderly individuals. (**A**) Melatonin and (**B**) cortisol serum concentrations. Data are shown as individual values with mean ± SE. Statistical analysis was performed using Student’s *t*-test. * indicates differences between groups. Melatonin: *p* = 0.0407 (institutionalized: *n* = 29; non-institutionalized: *n* = 25). Cortisol: *p* = 0.0039 (institutionalized: *n* = 28; non-institutionalized: *n* = 27).

As shown in Figure 3, institutionalized elderly individuals had markedly higher melatonin (43.1 ± 16.2 pg/mL) and cortisol (16.6 ± 2.5 ng/dL) concentrations than non-institutionalized peers living with family members (20.3 ± 6.9 pg/mL and 13.6 ± 2.9 ng/dL, respectively).

Melatonin and cortisol also showed large between-group effect sizes (d = 1.80 and d = 1.11, respectively), with high post hoc power (1–β = 0.99–1.00), reinforcing the robustness of the observed hormonal differences between institutionalized and non-institutionalized elderly individuals.

Table 2 presents the correlation analysis between melatonin and cortisol concentrations in institutionalized and non-institutionalized elderly individuals. When comparing the two groups, melatonin levels showed a weak, non-significant negative correlation (*r* = −0.3842, *p* = 0.2433), whereas cortisol concentrations showed a strong, statistically significant positive correlation (*r* = 0.8309, *p* = 0.0055). In the within-group analyses, no significant correlation between melatonin and cortisol was observed among non-institutionalized participants (*r* = −0.0439, *p* = 0.9107). In contrast, a moderate positive correlation was found among institutionalized elderly (*r* = 0.589, *p* = 0.0448).

**Figure 4:**
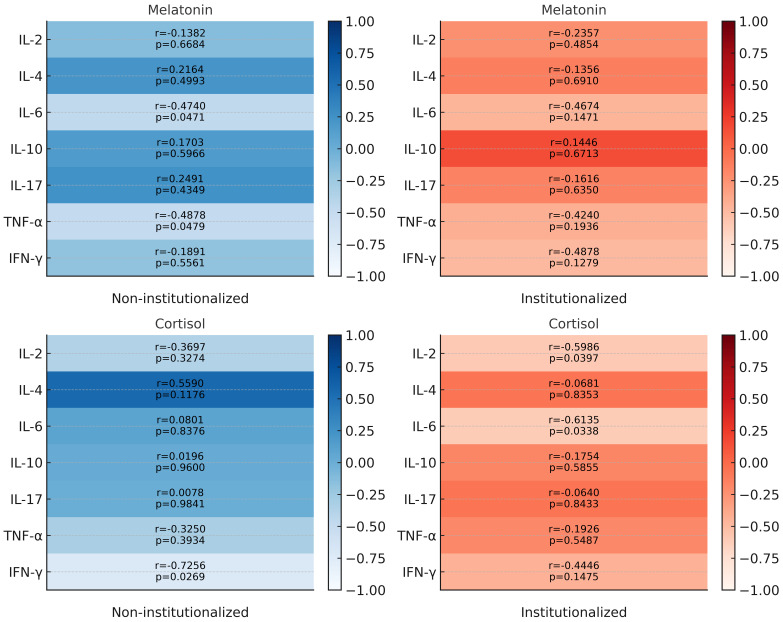
Correlation matrices between hormones and cytokines in institutionalized and non-institutionalized elderly individuals. Heatmaps showing Pearson correlation coefficients (r) and corresponding *p* values between cortisol or melatonin concentrations and serum cytokines (IL-2, IL-4, IL-6, IL-10, IL-17, TNF-α, and IFN-γ). The upper panels show melatonin correlations, and the lower panels show cortisol correlations. Statistical significance was considered at *p* < 0.05.

In Figure 4 (upper panels), melatonin did not exhibit significant correlations with cytokines in institutionalized elderly individuals (*p* > 0.05). In non-institutionalized older adults, however, melatonin showed negative correlations with IL-6 and TNF-α (*r* = −0.4674, *p* = 0.0471; *r* = −0.4878, *p* = 0.0479, respectively). In contrast, the lower panels indicate that cortisol presented negative correlations with IL-2 and IL-6 among institutionalized participants (*r* = −0.5986, *p* = 0.0397; *r* = −0.6135, *p* = 0.0338, respectively), as well as a negative correlation with IFN-γ in non-institutionalized elderly individuals (*r* = −0.7256, *p* = 0.0269).

## 4. Discussion

This study analyzes the serum levels of Th1, Th2, and Th17 cytokines, along with the hormones melatonin and cortisol, in elderly individuals living in institutions. The data indicate that institutionalization modulates both endocrine and immunological parameters, leading to alterations in hormonal balance and cytokine profiles when compared with non-institutionalized older adults.

Analysis of clinical and anthropometric characteristics revealed no significant differences between institutionalized and non-institutionalized elderly individuals. Both groups showed similar age distributions, sex proportions, and metabolic parameters, suggesting that the populations under study were homogeneous and comparable at baseline. Similar findings have been reported in other studies, which also observed comparable clinical and anthropometric characteristics between institutionalized and community-dwelling older adults [25,26].

The mean age of institutionalized participants was 78.2 ± 8.1 years, whereas that of the non-institutionalized group was 72.4 ± 7.5 years; however, this difference did not appear to influence the overall physiological or metabolic balance. The sex distribution was also comparable between groups, thereby minimizing the potential influence of sex-related hormonal variations on immune and endocrine outcomes [27,28].

Furthermore, body mass index, body weight, and waist circumference values were similar in both groups, indicating comparable nutritional and body composition profiles [25,26]. The absence of relevant differences in fasting glucose levels and in the prevalence of diabetes mellitus or hypertension supports the conclusion that the two populations had equivalent metabolic status and cardiovascular risk profiles [29,30].

These findings confirm that the institutionalized and non-institutionalized elderly participants had equivalent baseline conditions, thereby reducing confounding factors and enabling more accurate interpretation of subsequent analyses of cytokine patterns, cortisol, and melatonin levels. Given the group comparability confirmed across clinical, anthropometric, and metabolic parameters, the subsequent analysis focused on the cytokine network profile to characterize the immune status of the studied populations.

The observed cytokine patterns, including reductions in IL-2, IL-4, IL-17, and TNF-α, along with elevations in IL-6 and IFN-γ, suggest a selective remodeling of immunological pathways in institutionalized elderly individuals rather than a uniform suppression or activation of systemic inflammation. The decreases in IL-2 and IL-4 may reflect impaired T-cell proliferative capacity and attenuated Th2 regulatory activity, components well recognized within immunosenescence [3]. Likewise, the reductions in IL-17 and TNF-α are consistent with diminished Th17 effector responses and reduced monocyte–macrophage activation, patterns commonly described in aging [31].

In contrast, elevated IL-6 and IFN-γ levels align with the chronic low-grade inflammation characteristic of inflammaging. IL-6 is a pleiotropic cytokine that integrates metabolic, endocrine, and immune signals, and its increase has been associated with oxidative stress, neuroendocrine alterations, and imbalance in older adults [32,33,34]. Environmental and psychosocial stressors commonly found in long-term care facilities, such as reduced sunlight exposure, decreased social interaction, and disrupted sleep–wake cycles, can further enhance IL-6 production and sustain systemic inflammation. The concurrent rise in IFN-γ suggests compensatory Th1 pathway activation in response to persistent inflammatory or stress-related stimuli, which may preserve certain antimicrobial functions but also perpetuate tissue stress [35,36].

Overall, this pattern illustrates the coexistence of immunosenescence and inflammaging: adaptive immune pathways involving Th1, Th2, and Th17 responses decline, while innate inflammatory circuits remain active or become amplified. Thus, the cytokine signature observed here represents a selective proinflammatory shift driven predominantly by IL-6 and IFN-γ, whereas adaptive responses, reflected by lower levels of IL-2, IL-4, IL-17, and TNF-α, show functional decline. This combined profile shows age-related immune remodeling and suggests that institutionalization may further intensify these dysregulated networks.

Interestingly, IL-10 levels did not differ between groups, indicating a relative preservation of this essential anti-inflammatory pathway. IL-10 is fundamental for controlling IL-6 and IFN-mediated inflammatory activity and for maintaining immunological homeostasis [22]. The maintenance of IL-10, despite elevated proinflammatory mediators, may represent an adaptive compensatory mechanism aimed at limiting excessive inflammatory signaling under conditions of chronic stress [14,19]. This preservation aligns with evidence that, although aging is characterized by immunosenescence and age-related chronic inflammation (inflammaging), certain regulatory circuits, including IL-10-mediated feedback inhibition, remain functionally conserved [31]. Such maintenance of IL-10 signaling may help attenuate the increase in proinflammatory tone observed in institutionalized elderly, preventing a further escalation of innate immune activation and contributing to the partial retention of immunoregulatory balance.

Collectively, reduced levels of IL-2, IL-4, IL-17, and TNF-α, together with increased concentrations of IL-6 and IFN-γ and stable levels of IL-10, may reflect an altered immune network characteristic of elderly individuals living in institutional environments. These findings reinforce the notion that institutionalization amplifies neuroendocrine–immune imbalance and sustains a state of low-grade chronic inflammation, while residual regulatory mechanisms attempt to counteract excessive inflammatory activation.

This pattern, marked by reduced IL-2, IL-4, IL-17, and TNF-α in institutionalized individuals, indicates attenuation of Th1, Th2, and Th17 responses, reflecting the decline in adaptive immunity typical of immunosenescence. In contrast, elevated IL-6 and IFN-γ levels indicate a heightened proinflammatory environment, consistent with the concept of inflammaging. Meanwhile, the absence of significant differences in IL-10 levels suggests that compensatory anti-inflammatory regulation remains present but insufficient to counterbalance the sustained proinflammatory drive.

These findings highlight a complex interplay between neuroendocrine and immune regulation in elderly individuals, suggesting that institutionalization may influence both hormonal secretion patterns and cytokine network interactions. In the present study, both melatonin and cortisol concentrations were significantly higher in institutionalized elderly individuals than in those living with family members.

Evidence indicates that social isolation and reduced social engagement, common conditions in institutional environments, function as chronic psychosocial stressors that can disrupt hypothalamic–pituitary–adrenal (HPA) axis regulation. Diminished social support and persistent loneliness have been associated with altered diurnal cortisol secretion, reduced glucocorticoid feedback sensitivity, and heightened stress responsivity in older adults [37,38]. Such disruptions are known to amplify downstream inflammatory pathways, particularly those mediated by IL-6 and IFN-γ [39]. In this context, the elevated cortisol, IL-6, and IFN-γ levels observed in institutionalized elderly participants may reflect the physiological consequences of sustained social stress exposure, reinforcing the concept that institutionalization intensifies neuroendocrine–immune imbalance.

This concurrent elevation of two hormones with opposing circadian dynamics suggests a complex neuroendocrine adaptation to the environmental and psychosocial conditions associated with institutional living. Institutionalization, often accompanied by altered daily routines, increased exposure to artificial light, reduced sunlight, and persistent psychological stress, can disrupt circadian rhythms and prompt compensatory endocrine adjustments to maintain physiological homeostasis [40,41,42]. Elevated cortisol levels are consistent with chronic activation of the hypothalamic–pituitary–adrenal (HPA) axis, a classical feature of stress responses that has been associated with immunosenescence and increased production of proinflammatory cytokines in aging individuals [1,2]. Conversely, the increase in melatonin among institutionalized participants can reflect a compensatory regulatory response aimed at counterbalancing HPA axis hyperactivity. Melatonin is known to exert potent antioxidant and anti-inflammatory effects, acting as a modulator of immune cell activity and cytokine release through its interactions with MT1 and MT2 receptors expressed in lymphocytes and macrophages [17,19,43,44,45]. The moderate positive correlation observed between melatonin and cortisol levels in institutionalized individuals indicates a synchronized but dysregulated neuroendocrine adaptation to chronic stress.

When the hormonal findings are integrated with cytokine profiles, a clear pattern emerges linking endocrine modulation to immune activity. In this study, cortisol showed significant negative correlations with IL-2, IL-6, and IFN-γ, suggesting an inhibitory influence on both Th1 and Th17 immune pathways. These cytokines are key mediators of proinflammatory responses, and their suppression by cortisol is consistent with glucocorticoid-mediated immunomodulation. However, the simultaneous elevation of circulating cortisol and IL-6 observed among institutionalized individuals indicates that this regulatory feedback may be incomplete or desensitized, a hallmark feature of chronic low-grade inflammation, or inflammaging [1,46].

Melatonin, on the other hand, exhibited weak to moderate negative correlations with IL-6 and IFN-γ, supporting its anti-inflammatory role. Experimental studies have shown that melatonin can inhibit NF-κB activation, reduce IL-6 and IFN-γ secretion, and promote IL-10 expression, a cytokine crucial for controlling excessive immune activation [47,48]. Interestingly, in our cohort, IL-10 levels remained stable between groups, suggesting that anti-inflammatory pathways remain partially preserved despite the heightened proinflammatory environment.

Collectively, these data support the hypothesis that institutionalization exacerbates neuroendocrine–immune imbalance in elderly individuals. The co-occurrence of elevated cortisol, a compensatory rise in melatonin, and altered cytokine correlations suggests a state of physiological instability, in which anti-inflammatory mechanisms may attempt to counterbalance sustained immune activation. This integrated pattern aligns with the concept of inflammaging, characterized by chronic, low-grade inflammation driven by immune dysregulation, oxidative stress, and neuroendocrine maladaptation during aging [2,46].

Together, these findings emphasize the importance of environmental and psychosocial factors in shaping neuroendocrine and immune responses in older adults, reinforcing the need for interventions that mitigate chronic stress and preserve circadian and hormonal stability in institutional settings.

Overall, these findings highlight a neuroendocrine–immune imbalance and an altered cytokine network associated with institutionalization in elderly individuals. The clinical and anthropometric homogeneity observed between groups strengthens the internal validity of this study. It supports the interpretation that baseline clinical or metabolic disparities do not account for the differences observed in immunoendocrine parameters. Instead, they likely reflect the influence of environmental and psychosocial factors inherent to institutional living, such as changes in circadian rhythms, reduced social interaction, and chronic stress, which together contribute to neuroendocrine dysregulation and low-grade systemic inflammation during aging.

This study has several limitations. Although the sample was well characterized, the overall sample size was modest, reducing statistical power and limiting the robustness of subgroup analyses. The cross-sectional design further constrains the findings to associative rather than causal interpretations. Additionally, cortisol and melatonin were measured only once (8:00–10:00 a.m.), which standardizes sampling but does not capture their full circadian dynamics; multi-timepoint assessments are needed to elucidate how neuroendocrine rhythmicity shapes immune function in older adults.

Environmental determinants intrinsic to institutional living, such as light exposure, sleep–wake cycles, and physical activity, were not objectively monitored. Although routines were standardized and sunlight exposure was limited, participants did not engage in regular physical activity, and only 21% had formal mobility restrictions. Objective quantification of these factors in future studies may clarify the environmental modulation of neuroendocrine–immune pathways.

Despite standardized fasting, blood collection timing, and analytical blinding, cytokine measurements remain inherently variable and potentially influenced by unmeasured confounders. The single-center design may also limit generalizability, and multicenter approaches are warranted to validate and extend these findings across heterogeneous institutional settings.

Nevertheless, the study provides an integrated perspective by jointly evaluating neuroendocrine and immunological biomarkers, strengthening the understanding of low-grade chronic inflammation in aging. The clinically homogeneous groups enhance internal validity, and the observed interplay among cortisol, melatonin, and cytokine profiles suggests that institutionalization may modulate neuro–immune communication, potentially altering HPA axis and pineal signaling with downstream effects on systemic inflammatory networks.

**Scheme 2:**
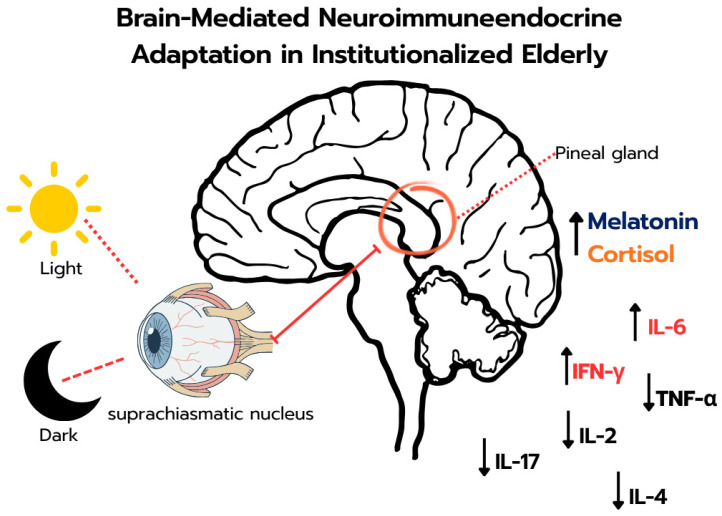
Neuroimmune–endocrine adaptation in institutionalized elderly individuals. Schematic illustration summarizing the observed neuroendocrine–immune imbalance in institutionalized elderly individuals.

Taken together, these findings identify potential targets for intervention—particularly approaches aimed at stabilizing circadian rhythms, mitigating psychosocial stress, and optimizing environmental light exposure—that may contribute to healthier neuroendocrine–immune regulation in institutionalized older adults. The integrated results of this study indicate that institutionalization is associated with a distinct neuroendocrine–immune imbalance, as summarized in Scheme 2. This imbalance reflects the combined effects of circadian disruption, concurrent elevations in cortisol and melatonin, and a modified proinflammatory cytokine profile, contributing to a chronic low-grade inflammatory state consistent with the concept of inflammaging [1,2,3].

In our cohort, institutionalized older adults showed elevated levels of both melatonin and cortisol. This hormonal pattern can represent a compensatory yet insufficient response to sustained environmental and inflammatory stressors. This dual elevation was accompanied by increased IL-6 and IFN-γ, cytokines associated with heightened inflammatory activity, alongside reduced IL-17, IL-2, and TNF-α, markers related to T-cell activation and adaptive immune coordination. These findings suggest that melatonin–cortisol interactions in institutionalized individuals contribute to a dysregulated immune milieu characterized by simultaneous proinflammatory signaling and impaired adaptive responses. Overall, our results demonstrate that institutionalization modulates both endocrine and immune parameters, resulting in measurable alterations in hormonal balance and in Th1, Th2, and Th17-associated cytokine profiles when compared with non-institutionalized older adults.

## 5. Conclusions

In conclusion, this study suggests that institutionalization can be associated with changes in neuroendocrine–immune dynamics in older adults. The higher concentrations of melatonin and cortisol, combined with the distinct cytokine patterns observed, can reflect a state of compensatory adaptation in which endocrine and immunological pathways attempt to maintain homeostasis under chronic environmental and psychosocial stressors. Although certain regulatory mechanisms, such as IL-10 activity, appear preserved, the overall profile is consistent with a shift toward increased proinflammatory signaling commonly described during aging.

## Figures and Tables

**Table 1:** Clinical characteristics of the patients under study.

Variables	Non-Institutionalized	Institutionalized
Number	29	32
Age (y)	74.4 ± 7.5	78.2 ± 8.1
Gender (% female)	12 (41.4%)	14 (43.7%)
Body Mass Index (kg/m^2^)	26.9 ± 5.2	25.4 ± 3.3
Body weight (kg)	57.0 ± 6.7	59.5 ± 11.3
Waist circumference (cm)	77.5 ± 1.7	73.2 ± 2.2
Glucose (mg/dL)	106.2 ± 14.6	109.8 ± 43.7
Diabetes mellitus	6/29 (20.7%)	7/32 (21.8%)
Hypertension	18/29 (62.1%)	21/32 (65.6%)

Data are expressed as mean ± SE or percentages (%).

**Table 2:** Correlation between melatonin and cortisol.

	Non-Institutionalized vs. Institutionalized
Melatonin	*r* = −0.3842
	*p* = 0.2433
Cortisol	*r* = 0.8309
	*p* = 0.0055 *
	**Melatonin × Cortisol**
non-institutionalized	*r* = −0.0439
	*p* = 0.9107
Institutionalized	*r* = 0.589
	*p* = 0.0448 *

Note: * statistical difference; *r* = Pearson correlation coefficient.

## Data Availability

The raw data supporting the conclusions of this article will be made available by the authors upon request. The data are not publicly available due to ethical and privacy restrictions associated with research involving human participants.
